# Reciprocal longitudinal effects between sense of school belonging and academic achievement: quasi-experimental estimates using United States primary school data

**DOI:** 10.3389/fpsyg.2024.1478320

**Published:** 2025-02-14

**Authors:** Chris Sakellariou

**Affiliations:** Nanyang Technological University, Singapore, Singapore

**Keywords:** sense of school belonging, academic achievement, reciprocal longitudinal effects, quasiexperimental methods, ECLS-K: 2011

## Abstract

**Introduction:**

This study investigates the bidirectional relationship between earlier sense of school belonging and later academic achievement in schoolchildren at grades 4 and 5 in US schools, using ECLS-K:2011 longitudinal data.

**Methods:**

Two alternative estimation methods were used, both addressing biases due to endogenous covariates.

**Results and discussion:**

The findings (sample size > 8,000 observations) provide strong evidence that (1) the dominant effect is from sense of school belonging to achievement, where lower bound effect sizes are substantially larger than those reported in correlational studies; and (2) in the opposite direction biases are small, and bias-corrected effect estimates are generally in line with the multiple regression estimates. The findings also provide suggestive evidence of larger effects for girls compared to boys in the direction from sense of school belonging to achievement scores. The study’s findings provide useful insights into the potential impact of school-based interventions.

## 1 Introduction

Social environments are increasingly recognized as influential channels for the diffusion of performance and learning, supported by a body of research (e.g., Dokuka et al., 2020; Stadtfeld et al., 2019; Smirnov and Thurner, 2017). Bandura’s Social Cognitive Theory (SCT) (Bandura, 1977) posits that learning is inherently social, occurring within a context where individuals observe and model behaviors, influenced by the consequences of those actions. This perspective underscores the role of social environments in shaping cognitive development. Complementary theories emphasize the evolutionary roots of social behavior, particularly within familiar and repeated interactions such as friendships (e.g., Pellegrini et al., 2002). These theories highlight the importance of repeated exposure and reciprocal relationships in fostering cooperative behaviors, aligning with Bandura’s framework of observational learning and social influence.

Research exploring the academic benefits of social integration in schools identifies two primary mechanisms. One emphasizes cognitive development through collaborative peer interactions, where students learn from each other and more skilled peers mentor those with less expertise (e.g., Wentzel et al., 2018; King et al., 1998). The other emphasizes the motivational and emotional support provided by friendships, which can positively influence attitudes toward learning and achievement (e.g., Hartup and Stevens, 1997). Baumeister and Leary (1995) refer to the inherent human need for belonging and social bonds, highlighting the detrimental effects of social disconnection on individuals’ well-being. Their work suggests that fostering feelings of belonging is essential for overall psychological health and stress management, portraying interpersonal attachment as a crucial aspect of human nature. Similarly, self-determination theory (Ryan and Deci, 2000) identifies three psychological needs—competency, autonomy, and relatedness—as fundamental drivers of human behavior. The need for relatedness specifically underscores the importance of forming supportive relationships in various contexts, including the school environment. This theory aligns with the concept of school connectedness, recognizing schools as primary settings where children seek and benefit from meaningful social connections.

The concept of social connectedness encompasses a range of experiences, including feelings of belonging and participation within social groups or communities (e.g., O'Rourke and Sidani, 2017). Academic performance is notably influenced by students’ feelings of connection to school. In particular, students who feel they belong at school are overall more motivated and engaged, have higher attendance, are less disruptive and distressed, achieve higher test scores, and complete school at higher rates (e.g., Allen and Bowles, 2012; Kuttner, 2023). By academic performance (or achievement) one refers to the extent to which a student has achieved his/her educational goals, such as grades and completion of educational degrees. Academic achievement is commonly measured through examinations, tests or continuous assessments. Hence, in the empirical literature, academic outcomes such as grades and standardized test scores, serve as common indicators of academic performance.

Research indicates that children who perceive a positive school environment tend to achieve higher grades, exhibit greater classroom motivation, possess higher academic self-efficacy, and show increased cognitive, emotional, and behavioral engagement at school (e.g., Crosnoe et al., 2004; Roeser et al., 1996). In addition to academic outcomes, students who feel connected to their school environments are less likely to engage in risky behaviors and demonstrate better social–emotional outcomes and psychological health (Suldo et al., 2009; Catalano et al., 2004; Resnick et al., 1997).

It is essential to recognize that while these studies demonstrate strong associations between school connectedness and various student outcomes, causation cannot be inferred. Furthermore, the directionality of these relationships remains complex, suggesting potential bidirectional influences between school connectedness and achievement. For instance, higher-achieving students could establish stronger relationships with teachers, positive relationships could enhance student achievement, or there may be a reciprocal reinforcement between school connectedness and academic success. Although indirect effects of belonging on academic motivation are acknowledged (e.g., Anderman, 2003; Goodenow and Grady, 1993), evidence of reciprocal effects between social connectedness and academic achievement remains limited. Furthermore, gender differences in the diffusion of academic performance through peer networks have been noted, with stronger peer influences observed among girls (Davies and Kandel, 1981).

To deepen our understanding, research must expand beyond associational studies and narrative reviews. Rigorous empirical investigations, employing quasi-experimental methods and innovative research designs are needed to quantify effect sizes and address potential endogeneity issues within the data. Moreover, exploring the reverse relationship can yield valuable insights into the bidirectional nature of these interactions.

In summary, social connections at school serve as critical conduits for the diffusion of performance and learning, shaped by theories of social cognitive learning and cooperative behavior. Expanding our understanding of social connectedness and integration requires robust empirical investigations, leveraging innovative methodologies to uncover causal relationships and bidirectional influences on academic achievement. External research underscores the transformative potential of supportive social environments in promoting positive youth development and academic success.

### 1.1 School belonging and academic achievement: overview of relevant literature

Students’ sense of school belonging is often defined as the extent to which students feel accepted, included, respected, and supported by peers and others within the school environment (e.g., Baumeister and Leary, 1995). School belonging is often referred to as school connectedness, and experts consider connectedness the most suitable synonym of school belonging. Other synonyms of belonging cited by experts include attachment, and engagement (Alink et al., 2023; Christenson et al., 2012). The most important indicators of school belonging according to experts are “inclusion,” “acceptance,” and “connection,” with “respect” and “positive relation to other students” also ranking high (Alink et al., 2023).

A substantial body of research has investigated the relationship between students’ sense of school belonging and their academic success in secondary education. A large part of this research reported in meta-analytic studies conducted over the past decade, overwhelmingly from correlational studies, have reported effect sizes related to the impact of social connectedness on academic outcomes. For instance, Moallem (2013) reported a modest effect size (bivariate correlation *r* = 0.22). Similarly, Korpershoek et al. (2020) and Wentzel et al. (2018) found small to moderate correlations between school belonging and academic achievement, particularly with standardized test scores.

A positive sense of belonging is important throughout a child’s schooling, particularly during periods of transition, such as from primary to secondary school. Students who report positive friendships and teacher-student relationships in primary school are more likely to report a positive sense of belonging in early secondary school (e.g., New South Wales Government, 2017). Studies on primary and middle-school children are few, especially studies focusing on the school connectedness-achievement dimension. Bond et al. (2007) examined associations between social relationships at grade 8 and educational achievement 2–4 years later. They found that having both good school and social connectedness in Year 8 was associated with the best outcomes (including high school completion) in later years. In contrast, the likelihood of completing school was reduced for those with either poor social connectedness, low school connectedness, or both. DeRosier and Lloyd (2011) tested whether social adjustment predicted academic outcomes above and beyond prior academic functioning, using school records and peer-, teacher-, and self-report measures for 1,255 third-grade children. Measures of social adjustment included social acceptance by and aggression with peers, while academic outcomes included math and reading grade point average. They used correlations and analyses of covariance (ANCOVAs) to test the degree to which social adjustment predicted academic outcomes. They found support for the causal model, i.e., that social adjustment contributed independently to the prediction of each area of academic adjustment. A more recent study used a large sample of Chinese primary and middle school pupils is by Yu et al. (2023). Using Pearson correlation analysis and ANOVA they found that the quality of personal relationships exhibited significant positive correlation with academic performance and that the quality of student-peer relationships was the most closely associated with academic achievement.

Two other studies (DiPrete and Jennings, 2012; Caemmerer and Keith, 2015) went beyond correlations, using US 1998–99 Early Child Longitudinal Study–Kindergarten Cohort data (as in this study); however, both did not investigate the relationship between a social connectedness measure (such as school belonging) and academic achievement but considered social skills a predictor of academic achievement in early childhood. DiPrete and Jennings (2012) supplemented OLS regressions with approaches that consider potential endogeneity issues and unobserved attributes, to find that social and behavioral skills (approaches to learning, self-control, and interpersonal skills) have substantively important effects on academic outcomes from kindergarten through fifth grade. Caemmerer and Keith (2015) used latent variable structural equation modeling to estimate bi-directional effects between social skills and student achievement from kindergarten to eighth grade. They found that the effects of students’ social skills and achievement are bi-directional, but the effects of students’ achievement on their later social skills are stronger than the effects of social skills on achievement.

### 1.2 Objectives of study

Earlier studies, some of which were cited in the previous sections, have effectively demonstrated that social connections influence various outcomes, including academic achievement. However, despite efforts in some studies to control for covariates, these methodologies often fail to fully account for unobservable differences among study participants, leading to biased effect size estimates. Addressing these biases due to endogeneity requires specialized techniques. Furthermore, few studies recognize that causality might not solely run from connectedness to academic achievement but could also operate vice versa. The correlation between students’ perceptions of connectedness and the error term can result from omitting relevant observed or unobserved covariates, or because both outcomes and connectedness perceptions are influenced by the same unobserved factors. For instance, students’ innate academic ability may correlate positively with academic achievement and also with connectedness perceptions. It is therefore important for the model to control for early cognitive ability (such as at kindergarten) and/or previous academic performance when available. Similarly, when investigating the opposite direction of the relationship (from earlier academic performance to later sense of connectedness), it is important to control for earlier measured social skills. Furthermore, measurement error in students’ assessment of connectedness can be an important source of bias due to endogeneity. Classical measurement error in a student connectedness construct, would lead to a downward-biased estimate of its effect on academic achievement.

While many empirical approaches like multivariate regression and propensity score matching do not recover causal effects, certain methodological approaches within non-experimental research designs can estimate quasi-causal effects, especially when longitudinal data are available. Using longitudinal data allows the researcher to identify changes over time in study participants’ lives (especially when studying developmental issues), which is important in assessing causation when combined with quasi-experimental methodologies (e.g., Rutkowski, 2016). Instrumental variables (IV) estimation is one such methodological approach that addresses biases associated with omitted variables, measurement error, and other sources of endogeneity of variables at the center of the investigation.

Therefore, this study intends to use an instrumental variables (V) approach as a main line of investigation, to estimate longitudinal bidirectional effects between sense of school belonging and academic achievement in primary school pupils in United States schools, after accounting for biases due to endogeneity of variables of interest. The objectives of the study are reflected in the following research questions:

#### 1.2.1 Research questions

RQ1: How large is the causal effect of earlier sense of school belonging on later academic achievement after accounting for biases due to potential endogeneity of sense of school belonging?
RQ2: Is there evidence of causal effects in the opposite direction?
RQ3: Are there gender differences in effect estimates in either direction?

This implementation is based on a methodological approach which used two alternative estimation methods, both of which account for biases due to endogenous covariates—IV estimation using instruments, supplemented with a method which does not rely on instruments.

The findings provide strong evidence that: (1) in the direction from sense of school belonging to achievement scores, bias-corrected effect estimates are substantially larger than multiple regression estimates, due to downward biases associated with the latter; and (2) in the opposite direction associated biases are small, and the bias-corrected effect estimates are generally in line with the multiple regression estimates.

In addition, in further assessing uncertainties associated with imperfect instruments, two other instrument-based approaches were used. One (Conley et al., 2012) allows for the construction of conservative confidence intervals for a mild violation of the exclusion restrictions, assuming plausible estimates of the direct effect of the instruments on the outcome. The second (Nevo and Rosen, 2012) derives confidence interval bounds in the presence of imperfect instruments utilizing assumptions about the sign and strength of the correlation between the imperfect instruments and the error term. The relevance of this line of investigation is that, with imperfect instruments, revised confidence intervals for effect estimates of interest may change certain conclusions derived from the main IV estimation.

## 2 Data and method

### 2.1 Data

The Early Childhood Longitudinal Study, Kindergarten Class of 2010-11 (ECLS-K:2011), conducted by the National Center for Education Statistics (NCES) between academic years 2010–2011 and 2015–2016 under the U.S. Department of Education’s Institute of Education Sciences (IES), is a comprehensive research initiative aiming to provide robust and comprehensive data for understanding children’s development and experiences during elementary school, examining how early experiences correlate with later development and learning outcomes. It has a broad scope, focusing on child development, early learning, and academic progress from kindergarten through fifth grade.

The participants in the ECLS-K:2011 study represent a diverse and nationally representative sample of children from both public and private schools, during the 2010–2011 academic year. This sample encompasses children from various socioeconomic backgrounds and racial/ethnic groups. The study also involves participation from the children’s parents, teachers, schools, and before-and after-school care providers. Fall and spring measures were collected from kindergarten to second grade, while for third to fifth grade only spring measures were collected.

Survey instruments include child assessment (language screener, reading, mathematics, science, executive function, and height and weight), parent interview, classroom teachers and special education teachers questionnaires (teacher and child level), and school administration questionnaires. The language screener in the child assessment was intended for children whose home language was not English. The components of the ECLS-K: 2011 assessments administered to children who spoke a language other than English at home depended on the children’s performance on a language screener.

The ECLS-K: 2011 data used in this study are ideal for investigating the reciprocal relationship between sense of school belonging and academic achievement. It contains items relating to academic achievement, sense of school belonging, and various items on teacher assessed children’s social skills. Information from the child, parent, and teacher questionnaires provides for a rich set of controls in modeling both directions of the relationship. It also contains assessments of early cognitive ability, as well as early social skills (both at kindergarten), which are important additional controls when the aim is to uncover causal effects in each direction.

#### 2.1.1 Concepts and measures

##### 2.1.1.1 Reading and mathematics scores

Assessment scores for each domain were calculated using IRT procedures. The *theta score* is an estimate of a child’s ability in each domain (reading, mathematics, and science), based on the child’s performance on the items actually administered. *Theta* scores range from −4 to 4, with higher scores indicating higher ability in the domain. The scores are approximately normally distributed. An IRT-based *overall scale score* was also calculated for each domain. It is an estimate of the number of items the child would have answered correctly if all the questions that were ever administered during the study for that domain had been administered to the child. Overall scale scores were calculated using a child’s *theta* score to predict the probability for each assessment item that the child would have given a correct answer for a particular item. The probabilities for all the items were summed to generate the overall scale score (U.S. Department of Education, 2018; U.S. Department of Education, 2019).

Assessment scores (standardized to mean 0 and SD of 1) in the domains of reading and mathematics were used in the analysis. Grade 5 scores were used as outcome in the direction from earlier sense of school belonging to later achievement, while grade 4 scores were used in the direction from earlier achievement to later sense of school belonging.

##### 2.1.1.2 Sense of school belonging

The Child questionnaire contains grade 4 and grade 5 students’ responses to questions on Peer Support at school (such as, “*classmates say they are my friend*”; “*classmates let me play with them*”; “*classmates make me happy*”; “*classmates make me feel better*”; “*classmates help when I am hurt*”); Loneliness (such as, “*I feel lonely at school*”; “*I feel left out/alone at school*”); Peer Victimization (such as, “*I was teased*”; “*others excluded me*”; “*I was pushed/shoved*”); and social anxiety at school (such as, “*I worry other kids do not like me*”; “*I worry what other kids think of me*”). In addition, grade 5 students responded to additional questions (how often: “*I feel like I fit in school*”; “*I enjoy school*”; “*I feel close to my classmates*”; “*I feel safe in school*”). In total, there are 16 items available for both grade 4 and grade 5 students, with an additional four items only for grade 5 students. These items, as a whole, exhibit high internal consistency, with internal consistency increasing the more items are included in the group. With the 16 common items, Cronbach’s alpha = 0.89; after adding the additional four items, Cronbach’s alpha = 0.92. School belonging continuous scales were constructed for grades 4 and 5 using principal component analysis and they were standardized (mean of 0 and SD of 1). Higher values in the index indicate higher sense of school belonging.

### 2.2 Method

#### 2.2.1 Accounting for biases due to endogeneity

IV estimation relies on the following assumptions: (a) “Relevance” of instruments, i.e., the instrument and the endogenous covariate are sufficiently correlated; (b) “Independence,” i.e., that there are no confounders of the association between the instrument/s and the outcome—an untestable assumption; and (c) “Valid Exclusion” of instruments, i.e., that the instrument must be independent of the outcome, after conditioning for additional covariates, i.e., the instrument can be validly excluded from the outcome equation.

An additional assumption, that of “monotonicity,” strictly interpreted requires that the level of the “treatment” received by a subject monotonically increases with the level of the instrument (e.g., Imbens and Angrist, 1994; Kennedy et al., 2019). Strict monotonicity is unlikely to hold in most cases, especially with a continuous outcome variable and an IV with several levels, as is the case here. Weak monotonicity (or stochastic monotonicity) is easier to satisfy since it requires a monotonic relationship between the different levels of the IV and the probability of treatment. de Caisemartin (2017) (see also Small and Tan, 2007) have shown that, overall, the 2SLS method is applicable in studies in which “defiers” could be present, under the weaker condition of stochastic monotonicity. This condition is more likely to hold if the IV has a strong first stage. In this case 2SLS estimates a Local Average Treatment Effect (LATE) as a weighted average marginal treatment effects, with an interpretation which is not as straightforward as that of the Average Treatment Effect.

Two sets of estimates were derived, using (1) Instrumental Variables estimation, exploiting overidentifying orthogonality conditions (main estimates), and (2) Kinky Least Squares (KLS) (Kiviet, 2022), which does not rely on availability of valid instruments (i.e., instruments are optional), but utilizes bounds on admissible degree of endogeneity of the potentially endogenous regressor, producing point estimates and confidence intervals for the entire grid of plausible endogeneity correlations. IV estimates were derived using sample replicate weights, while KLS estimates come unweighted. Effect estimates of the endogenous regressor on the outcome from KLS estimation can then be compared to the corresponding IV estimates. A consequence of obtaining KLS estimates from unweighted regressions is that standard errors are smaller and confidence intervals narrower; however, such standard errors and confidence intervals are not appropriate for inference.

In the context of IV estimation, in just-identified models, it is not possible to test the exclusion restrictions of individual instruments. Furthermore, in over-identified models, satisfying the Sargan/Hansen tests of overidentifying restrictions still relies on the validity of an initial set of untestable just-identifying orthogonality conditions, i.e., such tests presuppose validity of a number of external instruments equal to the number of endogenous regressors in the model. Hence validity of orthogonality conditions has to be justified using economic/theoretical arguments. Such arguments more often than not are disputed, in the absence of an empirical statistical test.

The KLS framework allows testing exclusion restrictions of individual instruments using plausible values of endogeneity correlations, i.e., testing the hypothesis, H_0_: *γ* = 0 in the model (y = Xß + Zγ + u), where Z is the instrument. However, while such a test can be formulated, acceptance of the null hypothesis at high *p*-values cannot be interpreted as necessarily supporting instrument validity, given the test lacks power to detect instrument invalidity (Kiviet, 2023). However, useful information can be derived if the test is used to detect cases where external instruments seem invalid due to low p-values, rather than claiming validity of the instruments when high p-values are derived for areas of plausible degree of endogeneity.

The methodological approach consists of the following steps: (1) deriving a plausible range of endogeneity correlations (point estimate and 95% CI); (2) testing the testable assumptions behind the IV method (i.e., relevance of instruments and overidentifying restrictions), and obtaining additional information on the likely (in)validity of exclusion restrictions for subsets of instruments using the *kinky least squares* (KLS) framework; and (3) deriving IV effect estimates using the proposed instruments, along with KLS estimates.

To derive a plausible range of endogeneity correlations, first, an Extended Regression Model (ERM) was estimated (using the Stata *eregress* module). ERMs is a Stata given name to a class of models that address frequently encountered complications such as endogenous covariates, sample selection and nonrandom treatment assignment (e.g., Heckman, 1976; Rubin, 1974). The estimates from this two-equation model mimic instrumental variables estimates, given the instrument/s used, which are assumed to be relevant and valid. In addition, a point estimate and associated 95% CI for the degree of endogeneity of the endogenous covariate (correlation of error terms between the two equations in the model) is derived.

#### 2.2.2 Proposed instruments for IV estimation

##### 2.2.2.1 From school belonging to achievement

In the ECLS-K: 2011, grade 4 and 5 teachers rated each student on a four-point scale (from “never” to “very often”) on various subscales of the Social Rating Scale, adapted from Social Skills Rating Scale (SSRS) of Gresham and Elliott (1990). Teacher ratings of children’s social skills are considered more reliable than parent ratings (e.g., DeRosier and Lloyd, 2011; Caemmerer and Keith, 2015). I used items from the interpersonal skills and self-control subscales. In the interpersonal skills subscale, teachers rated skills such as in getting along with others, expressing feelings, and showing sensitivity to the feelings of others. The self-control subscale rated the child’s ability to control behavior (such as respecting the property of other children). Four items were considered as instruments (single or in combination) for sense of school belonging at grade 4: “*expresses feeling and thoughts*”; “*sensitive to others*’ *feelings*”; “*comforts other children*”; and “*respects others’ property*.” Two items, which performed relatively better with respect to exclusion restrictions tests, were used in IV estimation—“*sensitive to others*’ *feelings*,” and “*respects others’ property*,” with admissible responses were, “Never,” “Sometimes,” “Often,” and “Very Often.” When constructing the instruments, responses for “Never” and “Sometimes” were combined, because of only a small number of respondents answered ‘Never’ in each item (generally less than 2%).

##### 2.2.2.2 From achievement to school belonging

Kindergarten, first-grade, and second-grade children were tested on two measures of executive function, assessing early cognitive abilities. Executive functions are interdependent processes that work together to regulate cognition, emotion, and behavior, facilitating learning in the classroom (e.g., Diamond, 2013). One is the Numbers Reversed test, a subtest of the Woodcock-Johnson III Tests of Cognitive Abilities (Woodcock et al., 2001), which assesses working memory. In the Numbers Reversed test, participants must repeat a series of random numbers backward, until they cannot remember the complete sequence or until they repeat it incorrectly. The other is the Dimensional Change Card Sort (DCCS) (Zelazo, 2006), assessing children’s cognitive flexibility. Children were asked to sort a series of 22 picture cards according to different rules—color or shape. First children are asked to sort by color (pre-switch trial), followed by the Shape Game (post-switch trial). If the child correctly sorted at least four of the six cards in the Shape Game, then he or she moved on to the Border Game, in which the sorting rule (by color or by shape) depended on whether the card had a black border around the edges. A single DCCS composite score was created by summing the post-switch score and the Border Game score. The Number reversed score and the composite DCCS score were assessed as instruments (single or in combination) for grade 4 achievement score as a predictor of sense of school belonging at grade 5.

Sense of school belonging is expected to be correlated with a child’s social skills in peer interactions (relevance of instruments), i.e., children with better social skills are expected to develop better social relationships and sense of belonging in school. In the opposite direction, children displaying stronger cognitive ability at kindergarten are expected to be academically later on.

In the direction from earlier sense of school belonging to later achievement, validity of the social skills-related instruments requires that they are uncorrelated with unaccounted characteristics (measured or unmeasured) that could also affect students’ achievement (other than through the instruments), after conditioning for covariates. However, children’s social skills may be correlated with characteristics such as family socioeconomic background, or children’s innate ability, i.e., children with better socioeconomic background, attending better quality schools, or more innately able students may acquire better social skills, hence performing better academically. It is therefore important to condition for early cognitive ability, along with socioeconomic status and school quality. The dataset contains information on a wide array of characteristics of children and their families, as well as measures of early cognitive ability (at kindergarten).

In the opposite direction, validity of the early cognitive ability as an instrument requires that cognitive ability at kindergarten age is uncorrelated with sense of school belonging at grade 5. Again, it is possible that children’s early ability may be correlated with characteristics which are also correlated with later sense of school belonging. For example, characteristics related to family background. This is because children of better educated-higher income parents, besides acquiring better cognitive skills, also acquired better social skills from the home environment, or attended better schools which provided a more conducive environment to foster social connections. Therefore, the model, besides family socioeconomic status and school characteristics, also conditions for (teacher rated) children’s early social skills (kindergarten).

To conclude, the (untestable) “validity” of instruments is not ensured, since one could think of scenarios in which the validity assumption does not hold; hence, instruments used in both directions are treated as imperfect instruments. However, after conditioning for crucial variables associated with these scenarios, conditional validity becomes more likely. With respect to validity of exclusion restrictions, information on their validity (specifically information which invalidates the exclusion restrictions) can be derived using the exclusion restriction tests that come with KLS estimation (given in the Supplementary material and discussed in the following section).

### 2.3 Model specification

In the model, the time interval in the longitudinal relationship between sense of school belonging and achievement is 12 months (from grade 4 to grade 5). This is because the information needed to implement model estimation is available for grades 4 and 5. Model specification was informed from reviewing the literature on likely exogenous determinants of achievement of schoolchildren. Such determinants include: (a) Personal factors (such as gender, race/ethnicity, health, cognitive abilities); (b) Family background (socioeconomic characteristics); and (c) School characteristics (such as school location, school type, school quality) (e.g., Costa et al., 2024). Possible determinants which are likely endogenous (such as student motivation and related self-constructs) were not included. In both directions of the relationship between grade 4 sense of school belonging and grade 5 achievement, the models (estimated by gender) control for student children’s personal characteristics, socioeconomic status, school characteristics, and school fixed effects. Personal characteristics include race/ethnicity (White, Black, Asian, American Indian/Alaska Native/Pacific Islander, and more than one race), location (city, suburb, town, rural), number of siblings, and having a disability. The socioeconomic status (SES) index combines parents’ education, parents’ occupational prestige, and household income. School characteristics include school type (public school, Catholic school, other private school), proportion of non-White students (0 to less than 25%, 25% to less than 50%, 50% to less than 75%, and 75%–100%), and index of school problems. The school problems index, ranging from negative to positive values (higher value indicating less problems/better quality schools) was derived using principal components analysis from the administrator’s questionnaire on frequency of problems such as bullying, student/teacher absenteeism, weapons in school, overcrowding, etc.

In the direction from grade 4 sense of school belonging to grade 5 achievement, the model also controls for early cognitive ability using the “Numbers Reversed W-Ability” score, measured at spring of kindergarten. Since one major potential source of endogeneity bias relates to children’s unobserved ability, it is important to control for early cognitive ability; not controlling for early unobserved ability is expected to result in biased estimates of connectedness effects. The “Numbers Reversed W-Ability” score measures children’s ability to manipulate information in working memory and to assess their attention and concentration skills. In the opposite direction, the model also controls for early (spring of kindergarten) teacher reported children’s social skills, based on items from the Social Skills Rating System (Gresham and Elliott, 1990). The interpersonal skills score ranges from 1 to 4, with a higher value indicating better interpersonal skills.

## 3 Results

### 3.1 Descriptive statistics

Table 1 contains the descriptive statistics on outcome variables and covariates by gender. Girls performed better in Reading, while boys performed better in Mathematics at both grades 4 and 5. Girls report stronger sense of school belonging as well as interpersonal skills while at kindergarten, as reported by teachers. Girls also scored higher on average in the “Numbers Reversed W-Ability” test while at kindergarten; however, the gender difference (while statistically significant), is modest. With respect to other covariates, no statistically significant gender differences appear in the estimation sample; one notable exception is that a substantially larger proportion of boys had a disability compared to girls.

**Table 1:** Weighted descriptive statistics by gender.

Characteristic	Male	Female	Male–Female Diff. [*p* value]
School grade			
Grade 5 Reading IRT Scale Score (standardized)	−0.020 (1.03)	0.075 (0.907)	[**0.000**]
Grade 4 Reading IRT Scale Score (standardized)	−0.038 (1.05)	0.100 (0.907)	[**0.000**]
Grade 5 Math IRT Scale Score (standardized)	0.059 (0.989)	−0.036 (0.955)	[**0.000**]
Grade 4 Math IRT Scale Score (standardized)	0.107 (1.00)	−0.067 (0.943)	[**0.000**]
Grade 5 School Belonging index (standardized)	0.005 (0.970)	0.077 (0.994)	[**0.001**]
Grade 4 School Belonging index (standardized)	−0.017 (0.987)	0.044 (1.00)	[**0.004**]
Child’s race/ethnicity			
White (%)	52.03 (49.96)	52.58 (49.94)	[0.608]
Black (%)	10.15 (30.20)	9.89 (29.85)	[0.687]
Hispanic (%)	28.73 (45.26)	27.36 (44.59)	[0.156]
Asian (%)	4.17 (19.98)	4.97 (21.74)	[0.070]
American Indian/Alaska Native/Pacific Islander (%)	1.31 (11.37)	1.29 (11.28)	[0.924]
More than one race (%)	3.51 (18.39)	3.90 (19.36)	[0.330]
Locale			
City (%)	28.78 (45.28)	30.94 (46.23)	[**0.031**]
Suburb (%)	41.15 (49.22)	39.18 (48.82)	[0.065]
Town (%)	11.19 (31.52)	10.56 (30.73)	[0.353]
Rural (%)	18.88 (39.14)	19.33 (19.49)	[0.602]
Number of siblings	1.64 (1.08)	1.62 (1.08)	[0.480]
Has disability	15.61 (33.66)	10.71 (28.51)	[**0.000**]
Socioeconomic status index (standardized)	−0.074 (0.750)	−0.077 (0.753)	[0.954]
School characteristics			
Public school (%)	92.91 (25.66)	92.21 (26.82)	[0.207]
Catholic school (%)	3.33 (17.93)	4.29 (20.26)	[**0.019**]
Other private school (%)	3.76 (19.02)	3.51 (18.40)	[0.530]
Proportion of non-White students: < 25%	33.91 (47.35)	34.80 (47.64)	[0.381]
Proportion of non-White students: 25–49%	23.42 (42.35)	23.51 (42.41)	[0.917]
Proportion of non-White students: 50–74%	17.15 (37.70)	16.04 (36.70)	[0.165]
Proportion of non-White students: 75–100%	25.53 (43.60)	25.65 (43.67)	[0.898]
School problems index (standardized)	−0.041 (0.998)	−0.018 (0.962)	[0.294]
Numbers reversed W-ability score (kindergarten)	449.7 (31.17)	451.9 (29.38)	[**0.001**]
Teacher reported interpersonal skills index (kindergarten)	2.92 (0.628)	3.13 (0.601)	[**0.000**]

Standard deviations in parentheses. Bold indicates statistical significance at the 5% level or lower.

### 3.2 Effect estimates

#### 3.2.1 Multiple regression (OLS) estimates

Estimated effects by gender, in both directions using OLS regressions (naïve model) are presented in Tables 2A,B. Both variables in the relationship between sense of school belonging and achievement are standardized continuous scales; hence effect sizes (d) report the change in the outcome from 1 SD increase in the variable of interest.

**Table 2:** (A) From social integration in school to achievement: OLS estimates by gender; (B) From achievement to social integration in school: OLS estimates by gender.

IRT scale score at grade 5 (stand.)	Reading		Mathematics	
	Males	Females	Males	Females
Grade 4: Sense of School Belonging (stand.)	**0.118** (0.017)	**0.078** (0.013)	**0.118** (0.016)	**0.070** (0.014)
Child’s race/ethnicity				
Black	**−0.237** (0.056)	**−0.239** (0.058)	**−0.511** (0.058)	**−0.523** (0.062)
Hispanic	−0.074 (0.046)	**−0.094** (0.031)	**−0.194** (0.045)	**−0.144** (0.039)
Asian	0.094 (0.084)	**0.126** (0.059)	**0.198** (0.076)	**0.194** (0.063)
Native American	**−0.163** (0.082)	−0.045 (0.095)	−0.279 (0.210)	0.015 (0.092)
Mixed Race/Ethnicity	0.064 (0.056)	0.080 (0.055)	−0.014 (0.074)	−0.094 (0.072)
Locale				
Suburb school (vs. City)	−0.031 (0.034)	**−0.059** (0.030)	−0.037 (0.032)	**−0.084** (0.035)
Town school (vs. City)	0.082 (0.077)	**−0.091** (0.044)	0.087 (0.059)	−0.078 (0.062)
Rural school (vs. city)	−0.061 (0.057)	**−0.092** (0.045)	−0.037 (0.044)	**−0.109** (0.044)
Number of siblings	**−0.059** (0.016)	**−0.044** (0.013)	−0.009 (0.016)	0.006 (0.016)
Has disability	**−0.363** (0.048)	**−0.352** (0.056)	**−0.399** (0.043)	**−0.388** (0.056)
SES (stand. index)	**0.253** (0.024)	**0.244** (0.017)	**0.277** (0.025)	**0.258** (0.020)
School characteristics				
Catholic school (vs. public)	**−0.099** (0.045)	0.001 (0.059)	**−0.228** (0.060)	**−0.140** (0.053)
Other private school (vs. public)	**−0.227** (0.078)	−0.010 (0.057)	**−0.299** (0.068)	**−0.202** (0.041)
25–50% non-White (vs. 0–25%)	0.025 (0.043)	0.077 (0.040)	0.027 (0.043)	0.029 (0.047)
50–75% non-White (vs. 0–25%)	−0.073 (0.051)	**−0.109** (0.049)	0.006 (0.053)	−0.081 (0.055)
>75% non-White (vs. 0–25%)	**−0.123** (0.059)	−0.044 (0.053)	−0.047 (0.072)	**−0.131** (0.061)
School problems (stand. index)	**0.046** (0.017)	**0.037** (0.016)	0.033 (0.018)	0.009 (0.014)
Numbers reversed ability score (Stand.)	**0.398** (0.020)	**0.363** (0.016)	**0.405** (0.019)	**0.421** (0.019)
Constant	**0.314** (0.058)	**0.261** (0.046)	**0.354** (0.069)	**0.205** (0.052)
*R* ^2^	0.38	0.371	0.454	0.435
*F*-statistic [*p* value]	143.4 [0.000]	66.0 [0.000]	102.7 [0.000]	106.5 [0.000]
*N*	4,859	4,677	4,858	4,676

Sense of school belonging at grade 5 (stand.)	Reading		Mathematics	
	Males	Females	Males	Females
Grade 4: IRT scale score (stand.)	**0.115** (0.021)	**0.101** (0.024)	**0.107** (0.023)	**0.093** (0.027)
Child’s race/ethnicity				
Black	**0.106** (0.053)	−0.003 (0.062)	**0.144** (0.054)	0.031 (0.064)
Hispanic	**0.208** (0.048)	**0.129** (0.052)	**0.213** (0.048)	**0.132** (0.053)
Asian	0.021 (0.072)	−0.096 (0.076)	0.018 (0.075)	−0.105 (0.074)
Native American	0.203 (0.136)	**−0.263** (0.097)	0.185 (0.133)	**−0.258** (0.096)
Mixed Race/Ethnicity	0.144 (0.098)	−0.115 (0.062)	0.167 (0.099)	−0.108 (0.082)
Locale				
Suburb school (vs. City)	−0.016 (0.043)	0.038 (0.041)	−0.017 (0.042)	0.036 (0.041)
Town school (vs. City)	0.009 (0.065)	**−0.248** (0.067)	0.004 (0.063)	**−0.251** (0.065)
Rural school (vs. city)	**−0.113** (0.051)	−0.098 (0.062)	**−0.130** (0.053)	−0.100 (0.061)
Number of siblings	**0.047** (0.017)	0.013 (0.020)	**0.042** (0.018)	0.006 (0.019)
Has disability	−0.091 (0.062)	**−0.291** (0.054)	−0.099 (0.065)	**−0.301** (0.051)
SES (stand. index)	0.002 (0.029)	**0.066** (0.025)	0.008 (0.029)	**0.071** (0.024)
School characteristics				
Catholic school (vs. public)	0.082 (0.070)	−0.081 (0.085)	0.081 (0.074)	−0.070 (0.085)
Other private (vs. public)	−0.175 (0.031)	−0.022 (0.091)	−0.174 (0.130)	−0.006 (0.090)
25–50% non-White (vs. 0–25%)	**−0.105** (0.050)	**−0.123** (0.055)	**−0.101** (0.048)	**−0.113** (0.054)
50–75% non-White (vs. 0–25%)	**−0.125** (0.059)	**−0.158** (0.072)	**−0.138** (0.059)	**−0.160** (0.070)
> 75% non-White (vs. 0–25%)	**−0.223** (0.059)	**−0.152** (0.069)	**−0.231** (0.059)	**−0.147** (0.068)
School problems (stand. index)	**0.053** (0.024)	**0.047** (0.024)	**0.055** (0.024)	**0.049** (0.024)
Social skills at kindergarten (Teacher-rated)	**0.217** (0.033)	**0.166** (0.035)	**0.218** (0.034)	**0.168** (0.036)
Constant	**−0.583** (0.123)	**−0.352** (0.146)	**−0.587** (0.124)	**−0.341** (0.146)
*F*-statistic [*p*-value]	0.065	0.058	0.062	0.058
	17.8 [0.000]	10.4 [0.000]	18.2 [0.000]	8.10 [0.000]
*N*	4,423	4,296	4,423	4,297

The model controls for grade 5 school fixed effects. Standard errors derived using 80 Jackknife replication weights in parentheses. Bold indicates statistical significance at the 5% level or lower.

The size of the effect of the variable of interest (grade 4 sense of school belonging, and grade 4 scale score in Reading/Mathematics) on academic outcomes (grade 5 achievement scale scores and grade 5 sense of school belonging) is small, at about 0.1 S.D. increase in the outcome for one S.D. increase in the predictor of interest. In the direction from sense of school belonging to academic scores, male estimates are somewhat larger than female estimates (difference statistically significant at the 5% level); in the opposite direction, the gender difference in effect estimates is not statistically significant. These effect sizes are representative of findings in several other empirical studies which rely on correlations (e.g., Korpershoek et al., 2020; Wentzel et al., 2018).

### 3.3 Results: from grade 4 sense of school belonging to grade 5 achievement scores

Tables 3A,B (top panel) report the second-stage IV estimates for the effect of sense school belonging on reading and mathematics standardized scale scores, along with coefficient estimates for other controls. First-stage results are given in Supplementary material. Using the combination of instruments, effect sizes from IV estimation are 3–5 times larger than the corresponding OLS estimates, and all differences are statistically significant. With respect to gender differences in effect sizes from IV estimation, the estimates for boys (*d* = 0.402; 95% CI [0.245, 0.560] on the reading score; and *d* = 0.354; 95% CI [0.183, 0.524] on the mathematics score) are smaller than the corresponding effect sizes for girls (*d* = 0.567; 95% CI [0.351, 0.784] on the reading score; and *d* = 0.535; 95% CI [0.292, 0.779] on the mathematics score); however, based on associated 95% confidence intervals, no clear statistical significance can be established for gender differences in effect estimates. The substantial difference between OLS and IV effect estimates is consistent with the strong endogeneity of the sense of school belonging construct. The Chi-sq test strongly rejects the null hypothesis that sense of school belonging is exogenous. In fact, the deviation between the OLS and the IV estimates increases as the (negative) endogeneity correlation estimate (degree of endogeneity of the sense of belonging construct) increases.

**Table 3:** (A) From social integration in school to reading achievement: IV effect estimates by gender; (B) From social integration in school to mathematics achievement: IV effect estimates by gender.

	Males		Females	
	IV: Second stage	KLS	IV: Second stage	KLS
A. Outcome: Reading IRT scale score at grade 5 (stand.)				
Grade 4: Sense of school belonging (stand.)	**0.402** (0.079)	**0.382** (0.023)	**0.567** (0.109)	**0.480** (0.035)
Child’s race/ethnicity				
Black	**−0.268** (0.062)		**−0.264** (0.082)	
Hispanic	−0.081 (0.047)		**−0.162** (0.041)	
Asian	0.079 (0.101)		**0.150** (0.072)	
Native American	−0.153 (0.098)		−0.044 (0.139)	
Mixed Race/Ethnicity	0.016 (0.058)		**0.171** (0.081)	
Locale				
Suburb school (vs. City)	−0.026 (0.041)		**−0.078** (0.037)	
Town school (vs. City)	0.091 (0.079)		0.019 (0.070)	
Rural school (vs. city)	−0.005 (0.073)		−0.001 (0.056)	
Number of siblings	**−0.065** (0.016)		**−0.048** (0.016)	
Has disability	**−0.251** (0.057)		**−0.179** (0.064)	
SES (stand. index)	**0.242** (0.026)		**0.188** (0.030)	
School characteristics				
Catholic school (vs. public)	**−0.095** (0.042)		0.055 (0.87)	
Other private (vs. public)	−0.062 (0.084)		0.053 (0.087)	
25–50% non-White (vs. 0–25%)	0.060 (0.048)		**0.168** (0.055)	
50–75% non-White (vs. 0–25%)	0.002 (0.064)		−0.040 (0.063)	
> 75% non-White (vs. 0–25%)	−0.062 (0.079)		0.056 (0.069)	
School problems (stand. index)	0.035 (0.020)		0.011 (0.023)	
Numbers reversed ability score at kindergarten	**0.375** (0.021)		**0.310** (0.022)	
Constant	**0.253** (0.073)		**0.186** (0.063)	
*F*-statistic [*p* value]	140.1 [0.000]		77.4 [0.000]	
First stage: Sense of School Belonging (standardized)				
Instruments:							
*Sensitive to others’ feelings*	**0.185** (0.021)	**0.131** (0.029)					
*Respects others’ property*	**0.137** (0.028)	**0.144** (0.043)					
Relevance of excluded instrument:				
Shea Partial R-sq. of excluded instrument	0.047		0.026	
Montiel Olea – Pflueger weak instrument test:				
Effective F-statistic:	69.4		32.6	
Critical values for 5% worst-case bias:	7.22		7.07	
Overidentification test of all instruments:				
Hansen’s J statistic [*p* value]	0.116 [0.733]		0.142 [0.706]	
Test of endogeneity of social integration:				
Chi-sq. statistic [*p* value]	16.9 [0.000]		31.1 [0.000]	
*N*	4,103	4,103	4,011	4,011
B. Outcome: Math IRT Scale score at grade 5 (stand.)				
Grade 4: sense of school belonging (stand.)	**0.354** (0.086)	**0.348** (0.020)	**0.535** (0.122)	**0.461** (0.034)
Child’s race/ethnicity				
Black	**−0.552** (0.065)		**−0.537** (0.079)	
Hispanic	**−0.201** (0.045)		**−0.204** (0.046)	
Asian	**0.187** (0.083)		**0.230** (0.097)	
Native American	−0.290 (0.209)		−0.010 (0.140)	
Mixed Race/Ethnicity	−0.068 (0.085)		−0.025 (0.104)	
Locale				
Suburb school (vs. City)	−0.032 (0.032)		**−0.097** (0.039)	
Town school (vs. City)	0.069 (0.063)		0.015 (0.082)	
Rural school (vs. city)	0.010 (0.048)		−0.015 (0.054)	
Number of siblings	−0.014 (0.015)		0.005 (0.019)	
Has disability	**−0.320** (0.053)		**−0.215** (0.070)	
SES (stand. index)	**0.270** (0.024)		**0.206** (0.028)	
School characteristics				
Catholic school (vs. public)	**−0.216** (0.063)		−0.082 (0.068)	
Other private (vs. public)	**−0.255** (0.069)		−0.138 (0.076)	
25–50% non-White (vs. 0–25%)	0.061 (0.047)		0.113 (0.062)	
50–75% non-White (vs. 0–25%)	0.086 (0.065)		−0.015 (0.069)	
> 75% non-White (vs. 0–25%)	0.016 (0.086)		−0.044 (0.074)	
School problems (stand. index)	0.018 (0.019)		−0.016 (0.018)	
Numbers reversed ability score at kindergarten	**0.385** (0.021)		**0.370** (0.022)	
Constant	**0.314** (0.076)		0.114 (0.067)	
*F*-statistic [*p* value]	99.0 [0.000]		78.4 [0.000]	
First stage: sense of School Belonging (standardized)				
Instruments:				
*Sensitive to others’ feelings*	**0.185** (0.021)		**0.132** (0.029)	
*Respects others’ property*	**0.136** (0.028)		**0.145** (0.043)	
Relevance of excluded instrument:				
Shea Partial R-sq. of excluded instrument	0.047		0.026	
Montiel Olea—Pflueger weak instrument test:				
Effective *F*-statistic:	69		32.9	
Critical values for 5% worst-case bias:	7.16			
Overidentification test of all instruments:				
Hansen’s J statistic [p-value]	0.059 [0.809]		0.390 [0.532]	
Test of endogeneity of social integration:				
Chi-sq. statistic [*p*-value]	12.4 [0.000]		27. [0.000]	
*N*	4,101	4,101	4,010	4,010

The model controls for grade 5 school fixed effects. Standard errors derived using 80 Jackknife replication weights in parentheses. Bold indicates statistical significance at the 5% level or lower.

The alternative (KLS) estimates are of similar size. They were derived using the point estimate of the endogeneity correlation and come with narrower confidence intervals. Therefore, from the associated 95% confidence intervals (derived using underestimated standard errors, since KLS estimates are unweighted), the gender difference in effect sizes is statistically significant. The top part of Graph 1 in the Supplementary material (and likewise for other models) shows graphically the effect estimates for the entire grid of plausible endogeneity correlations, along with the (unweighted) IV estimate. The KLS estimates overlap with the confidence interval of the IV estimate for the entire range (95% CI) of the plausible endogeneity correlations.

First-stage tests for relevance of instruments (weak instrument tests) are given in the bottom panel of the results tables. The test proposed in Montiel Olea and Pflueger (2013), an extension of the test Stock and Yogo (2005) for weak instruments in linear IV regression is reported, along with the partial R-sq of excluded instruments. There is no indication that the instrument set is weak. From the values of the effective F-statistics and associated weak instrument tests, worst-case bias due to weak instruments is much lower than 5%.

The overidentification test of the instrument set (Hansen’s J) indicates that the null hypothesis that the overidentifying restrictions are valid is accepted in all cases at *p* values higher than 0.5. However, in the case of two instruments and one endogenous regressor, the interpretation of such validity of the overidentifying restrictions is that one of the two instruments in the set is validly excluded, while the other is untested and assumed to be valid. Furthermore, overidentification tests can have low power (e.g., Kripfganz and Kiviet, 2021). Therefore, further scrutiny of the validity of exclusion restriction for each instrument is needed.

Information on the possible invalidity of exclusion restrictions of instruments is presented graphically in graphs 1–2 by gender, utilizing the KLS framework. The top part of each graph depicts the effect estimates across the grid of postulated degrees of endogeneity (derived from KLS regressions), along with the corresponding IV estimate of the effect of the variable of interest on the outcome. The bottom part graphically depicts instrument exclusion restriction tests for plausible values of degree of endogeneity of the endogenous regressor (endogeneity correlations). The vertical axis depicts the associated *p* value for accepting the hypothesis of valid exclusion of the instrument. The instrument is assessed by comparing the point estimate and 95% CI of the endogeneity correlation to the point estimate and 95% confidence interval for the degree of endogeneity compatible with valid exclusion give in the exclusion restriction test. Plausible endogeneity correlations (point estimates and 95% CI) were derived as described in the methodology section. In all cases, point estimates of endogeneity coefficients are negative and significant, and are larger in the female sample. In all cases, the hypothesis of valid exclusion is accepted at *p* values ranging from 0.65 to 0.95, for the point estimate of plausible endogeneity correlations; this is the case for each instrument separately, as well when used as a combination. Therefore, while this does not ensure instrument validity (instrument validity stays untested), the exclusion restriction tests indicate that neither of the two external instruments seem invalid.

### 3.4 Results: from grade 4 achievement scores to grade 5 sense of school belonging

Second-stage IV estimates for the effect of grade 4 achievement score on grade 5 sense of school belonging are given in Tables 4A,B (and detailed first-stage results in Supplementary material). In the male sample, IV estimates of the effect of earlier achievement on later sense of school belonging (*d* = 0.145 for Reading and *d* = 0.156 for Math) are only slightly larger than the corresponding OLS estimates (*d* = 0.115 for Reading and *d* = 0.107 for Math); based on comparison of 95% confidence intervals, differences in the two sets of estimates are not statistically significant. In the female sample, the IV estimates are somewhat larger (*d* = 0.196 for Reading and *d* = 0.181 for Math), are statistically different from the corresponding OLS estimates (*d* = 0.101 for Reading and *d* = 0.093 for Math) only at the 10% level. The alternative (KLS) estimates are also of similar size. Based on Chi-sq. endogeneity tests, in the male sample, exogeneity of the achievement score variable is accepted with an associated *p* value of 0.477 for Reading and 0.198 for math; in the female sample, exogeneity is marginally rejected for Reading (*p* value = 0.047) and marginally accepted for Math (*p* value = 0.057).

**Table 4:** (A) From reading achievement to social integration in school: IV effect estimates by gender; (B) From mathematics achievement to social integration in school: IV effect estimates by gender.

	Males		Females	
	IV: Second stage	KLS	IV: Second stage	KLS
A. Outcome: sense of school belonging at grade 5 (stand.)				
Grade 4: Reading IRT scale score (stand.)	**0.145** (0.051)	**0.150** (0.019)	**0.196** (0.053)	**0.187** (0.020)
Child’s school/ethnicity				
Race/Ethnicity: Black	**0.138** (0.059)		−0.019 (0.064)	
Race/Ethnicity: Hispanic	**0.206** (0.047)		**0.123** (0.054)	
Race/Ethnicity: Asian	0.057 (0.074)		−0.123 (0.077)	
Race/Ethnicity: Native American	0.210 (0.143)		**−0.310** (0.102)	
Mixed Race/Ethnicity	0.140 (0.102)		−0.117 (0.087)	
Locale				
Suburb school (vs. City)	−0.009 (0.046)		0.048 (0.047)	
Town school (vs. City)	0.022 (0.077)		**−0.230** (0.078)	
Rural school (vs. city)	**−0.108** (0.055)		−0.105 (0.067)	
Number of siblings	0.037 (0.019)		0.021 (0.020)	
Has disability	−0.082 (0.066)		**−0.248** (0.065)	
SES (stand. index)	−0.012 (0.038)		0.028 (0.028)	
School characteristics				
Catholic school (vs. public)	0.087 (0.076)		−0.083 (0.087)	
Other private (vs. public)	−0.187 (0.134)		−0.014 (0.093)	
25–50% non-White (vs. 0–25%)	**−0.123** (0.054)		**−0.122** (0.060)	
50–75% non-White (vs. 0–25%)	**−0.158** (0.065)		−0.130 (0.074)	
> 75% non-White (vs. 0–25%)	**−0.225** (0.065)		−0.126 (0.075)	
School problems (stand. index)	0.039 (0.027)		0.040 (0.025)	
Social Skills at kindergarten (Teacher-rated)	**0.200** (0.036)		**0.145** (0.036)	
Constant	**−0.511** (0.126)		**−0.332** (0.156)	
*F*-statistic [*p* value]	12.3 [0.000]		7.52 [0.000]	
First stage: Reading score (standardized)				
Instruments:				
*Numbers Reversed ability score at kindergarten*	**0.376** (0.021)		**0.316** (0.017)	
*Dimensional Change Card Sort at kindergarten*	**0.083** (0.011)		**0.078** (0.010)	
Relevance of excluded instrument:				
Partial R-sq. of excluded instrument	0.179		0.161	
Montiel Olea—Pflueger weak instrument test:				
Effective F-statistic	477.7		422.1	
Critical values for 5% worst-case bias	6.64		7.18	
Overidentification test of all instruments:				
Hansen’s J statistic [*p* value]	0.117 [0.733]		0.052 [0.820]	
Test of endogeneity of social integration:				
Chi-sq. statistic [*p* value]	0.505 [0.477]		3.98 [0.046]	
*N*	3,707	3,707	3,708	3,708
B. Outcome: Sense of school belonging at grade 5 (stand.)				
Grade 4: Math IRT Scale Score (stand.)	**0.156** (0.055)	**0.162** (0.021)	**0.181** (0.051)	**0.190** (0.022)
Child’s race/ethnicity				
Black	**0.195** (0.066)		0.043 (0.067)	
Hispanic	**0.220** (0.049)		**0.127** (0.053)	
Asian	0.059 (0.077)		−0.142 (0.076)	
Native American	0.192 (0.139)		**−0.302** (0.096)	
Mixed Race/Ethnicity	0.172 (0.104)		−0.102 (0.086)	
Locale				
Suburb school (vs. City)	−0.012 (0.045)		0.046 (0.047)	
Town school (vs. City)	0.012 (0.073)		**−0.235** (0.075)	
Rural school (vs. city)	**−0.130** (0.055)		−0.108 (0.066)	
Number of siblings	0.033 (0.019)		0.011 (0.019)	
Has disability	−0.076 (0.070)		**−0.261** (0.062)	
SES (stand. index)	−0.012 (0.038)		0.037 (0.028)	
School characteristics				
Catholic school (vs. public)	0.085 (0.082)		−0.064 (0.086)	
Other private (vs. public)	−0.182 (0.132)		0.015 (0.090)	
25–50% non-White (vs. 0–25%)	**−0.116** (0.051)		−0.101 (0.057)	
50–75% non-White (vs. 0–25%)	**−0.171** (0.064)		**−0.135** (0.070)	
> 75% non-White (vs. 0–25%)	**−0.228** (0.065)		−0.114 (0.074)	
School problems (stand. index)	0.042 (0.027)		0.045 (0.025)	
Social Skills at kindergarten (Teacher-rated)	**0.196** (0.037)		**0.145** (0.037)	
Constant	**−0.520** (0.126)		**−0.306** (0.156)	
*F*-statistic [*p* value]	12.0 [0.000]		7.67 [0.000]	
First stage: Reading score (standardized)				
Instruments:				
*Numbers Reversed ability score at kindergarten*	**0.343** (0.017)		**0.344** (0.018)	
*Dimensional Change Card Sort at kindergarten*	**0.085** (0.010)		**0.077** (0.010)	
Relevance of excluded instrument:				
Partial R-sq. of excluded instrument	0.195		0.181	
Montiel Olea—Pflueger weak instrument test:				
Effective *F*-statistic	496.1		508.8	
Critical values for 5% worst-case bias	7.99		5.8	
Overidentification test of all instruments:				
Hansen’s J statistic [*p* value]	0.068 [0.794]		0.018 [0.894]	
Test of endogeneity of social integration:				
Chi-sq. statistic [*p* value]	1.66 [0.198]		3.62 [0.057]	
*N*	3,708	3,708	3,708	3,708

The model controls for grade 5 school fixed effects. Standard errors derived using 80 Jackknife replication weights in parentheses. Bold indicates statistical significance at the 5% level or lower.

The instrument set is strong, and the effective F-statistic is larger than in the direction from sense of school belonging to achievement. The overidentification test of the instrument set accepts the null hypothesis that the overidentification restrictions are valid, with a p-value for Hansen’s J test between 0.7 and 0.9. Graphs 3–4 assess possible invalidity of exclusion restrictions of instruments. Here, the dominant instrument is the “Number Reversed” score at kindergarten, which is associated with a relatively narrower range of acceptable endogeneity correlations compared to the “Dimensional Change Card” Sort score at kindergarten. Plausible endogeneity correlation estimates are negative, but generally small and statistically insignificant. In all cases, the point estimate and entire 95% CI of estimated endogeneity correlations lie inside the range of endogeneity of instrument compatible with valid exclusion. Again, while there is no assurance of instrument validity, the exclusion restriction tests do not suggest that the external instruments are invalid.

### 3.5 Other covariates

The measure of early ability (“Numbers Reversed” score) at kindergarten (which assesses ability to manipulate information in working memory and to assess their attention and concentration skills), is a strong predictor of later academic achievement. One SD increase in the “Numbers Reversed” score at kindergarten is associated with 0.3–0.4 SD higher achievement score at grade 5. Significant associations were also found with respect to race/ethnicity; being Black or Hispanic is negatively associated with achievement, compared to being White, while the opposite is the case for children identified as Asian. Socioeconomic status exhibits a strong positive association with achievement, while having a disability is negatively associated with achievement. In the reverse direction, the early, teacher-reported, measure of social skills exhibits a strong association with later sense of school belonging. Being Hispanic is positively associated with school belonging perceptions, and this is also the case for Black males. Finally, sense of school belonging is negatively associated with the proportion of non-White students at school (and more so for boys).

### 3.6 Further analysis

Given that overidentifying restriction tests with two instruments and one endogenous covariate leaves one instrument untested, one could look for significant differences in IV estimates when using one instrument at a time; such differences in estimates likely indicates that at least one of the instruments is invalid. In the direction from sense of school belonging to achievement, the two instruments are, “*sensitive to others’ feelings*,” and “*respects others’ property*.” IV estimates using each instrument alone (Supplementary material) are very similar to the main estimates. In the opposite direction, the dominant instrument is the “*Numbers Reversed*” *score*, hence the effect estimate mostly reflects this instrument. We would therefore want to look for large differences in effect estimates when the other, relatively weaker instrument (“*Card Sort*” *score*) is used (Supplementary material). Again, differences in effect estimates are modest, and earlier conclusions stand.

Since the independence assumption associated with instrument validity is not testable, associated uncertainties with respect to effect size estimates can be explored in the context of “imperfect” instruments. Two frequently used approaches are by Nevo and Rosen (2012) and Conley et al. (2012). The first relies on weaker assumptions than instrument exogeneity (specifically, assumptions about the sign of the correlation of the instruments with the error term), to derive effect size bounds and confidence intervals in the presence of imperfect instruments. The second utilizes information or assumes a plausible range of the direct effect of an instrument on the outcome, to construct conservative confidence intervals for mild violation of exclusion restrictions.

Supplementary material contain the results from implementing Nevo and Rosen (2012). In the direction from sense of school belonging to achievement, lower bound confidence intervals are narrower than upper bound confidence intervals, hence more informative. Using lower bounds, IV effect estimates are at least double the OLS estimates for both girls and boys. Upper bound estimates are subject to substantial uncertainty (much wider confidence intervals). This is consistent with the confidence intervals from KLS regressions for increasingly larger negative endogeneity correlation (top of graphs 1–2). Larger effect estimates (resulting from negative and increasing degree of endogeneity correlations), are associated with increasingly wider confidence intervals. In the reverse direction of the relationship, bounds are narrower, confirming the conclusion that effect sizes are modest, with confidence intervals containing the OLS estimates. When comparing effects by gender in the main direction by comparing (the wider) 95% confidence intervals, the conclusion that effects for girls are larger does not survive, since confidence intervals overlap for both academic outcomes.

To implement, Conley et al. (2012) estimates of the direct effect of each instrument on the outcome was derived from IV regressions in which only one of the two instruments is used, while the second is entered an additional regressor on the outcome and vice versa. Point estimates and conservative confidence intervals were derived using these estimates of direct effects of each instrument on the outcome (which were not statistically significant in all cases, but not zero). The results (given in Supplementary material) with respect to the lower bound confidence intervals, are consistent with the results of Nevo and Rosen (2012) in the main direction of the relationship between sense of school belonging and achievement, and for both lower and upper bounds in the reverse direction of the relationship.

Finally, some of the model covariates besides the variable of interest, notably school related covariates, are potentially endogenous. When relying on conditional independence of instruments, covariates measured later than the endogenous covariate/treatment of interest should not be confounded by unobservables, and the instrument should not directly affect any variables in the model measured after the endogenous covariate/treatment (other than through the endogenous covariate of interest) (e.g., Deuchert and Huber, 2017). For example, in our case, in in the reverse direction of the relationship (from academic achievement to sense of belonging), early cognitive ability (measured at kindergarten), could influence later decisions about the characteristics of school the child will later attend. The robustness of the results are assessed using a model which omits other potentially endogenous covariates (Supplementary material). Effect size estimates and related test statistics are consistent to those from the base estimates in both directions of the relationship.

## 4 Discussion and conclusions

This study was motivated by gaps in the existing research on how various conceptualizations of students’ sense of social integration (in our case sense of belonging in school), relate causally to academic achievement. Existing literature from meta-analytic and other studies primarily relies on bivariate correlations or multiple regressions to estimate effect sizes, occasionally controlling for some participant differences. However, it overlooks the role played by unobservables and associated biases in estimating effects in both directions of the relationship between sense of belonging in school and academic achievement.

To address this, the study combined the use of longitudinal data on United States primary school children from kindergarten to fifth grade, with a quasi-experimental strategy to explore biases in effect estimates linked to potential endogeneity of covariates. IV estimation was supplemented with alternative methodologies which account for biases due to endogeneity, while sensitivity analysis explored the extent of uncertainly in effect size estimates.

In the direction from earlier sense of school belonging to later academic achievement, failing to consider the endogeneity of sense of school belonging led to significant underestimation of effect sizes. These biases seem more pronounced among female students, based on larger estimated endogeneity correlations in the female relative to the male sample, resulting in larger effect sizes for girls. This suggests that the influence of peer interactions on reading and math literacy, is likely stronger for girls. However, when comparing effect sizes by gender by comparing 95% confidence intervals, while from the main IV estimation one can conclude that estimated effects for girls are statistically marginally larger than for boys, this is not the case when using 95% confidence intervals when assuming imperfect instruments (from Nevo and Rosen IV regressions). Reciprocal effects were also established; however, effect sizes from IV regressions, are only slightly larger and not statistically different from those derived from multiple (OLS) regressions.

The findings show that measured academic achievement, which reflects students’ literacy, is less subject to endogeneity biases compared to students’ perceptions of social integration in the school environment. It may very well be a measurement/misreporting error story. In the direction from sense of belonging to academic achievement, self-reported perceptions related to children’s sense of belonging in school are likely more mismeasured/misreported compared to measures of academic achievement. If measurement/misreporting error in student responses on their sense of belonging is random in nature (not systematic), OLS estimates of the effect of social integration on academic achievement will be downward biased.

The finding that the influence of peer interactions on reading and math literacy may be stronger for girls (while subject to uncertainty—hence only suggestive), is supported by some earlier studies which have noted gender differences in the influence of peers on educational aspirations and achievement, with females often exhibiting stronger responses to social influences, reflecting gender disparities in social and behavioral skills acquisition and educational outcomes (e.g., Davies and Kandel, 1981; Han and Li, 2009). Such findings are consistent with social psychology theories that females are more influenced by peers (e.g., Minton and Schneider, 1980).

The study’s findings provide useful insights into the potential impact of school-based interventions. Academic success is significantly shaped by heritable traits and individual-specific environmental factors (e.g., Rimfeld et al., 2018). Since these factors do not typically respond to policy interventions, fostering students’ social connections within the school environment could lead to measurable outcomes. These interventions could focus on student groups that vary in their social connections within the school, as well as their academic performance. For example, observant teachers could pair/group students based on teachers’ criteria, rather than on students’ preferences when students are assigned class assignments, group projects, etc., given that students would likely prefer to interact with peers who share similar interests and/or peers with whom they have established social bonds.

It is therefore important that education authorities in coordination with school principals invest time and resources to programs intended to promote social connection at a school and classroom level. Toward this goal, they need to train teachers to be aware of the role they can play in identifying at risk students, such as those who may have limited social skills, are shy, seem to be rejected by their peers, or students under stress or exhibiting signs of mental illness. It is also important that teachers build social connections with at risk students and students in general. Finally, schools should track and measure social connection by conducting School Climate Surveys, which can inform regarding students’ social connectedness.

This study’s strengths lie in the combination of longitudinal data with quasi-experimental methods to go beyond establishing associations between a measure of student’s social integration in school and academic achievement and inform on effect size bounds in each direction of the relationship. Thus, in a large sample of United States primary school children, it establishes that the dominant effect is from sense of school belonging to achievement, where lower bound effect sizes are substantially larger than those reported in the literature which is based on correlational studies. The findings are also suggestive of larger effect sizes for girls compared to boys, at least in the main direction of the relationship. A weakness of the study is that, after considering uncertainties related to using imperfect instruments, upper bound effect size estimates and confidence intervals in the direction from sense of school belonging to achievement are wide—hence not very informative.

## Data Availability

Publicly available datasets were analyzed in this study. This data can be found here: https://catalog.data.gov/dataset/early-childhood-longitudinal-study-kindergarten-class-of-2010-11-3fa4e.
