# Supplementary material for: Reciprocal longitudinal effects between sense of school belonging and academic achievement: quasi-experimental estimates using United States primary school data
Source: Front Psychol. 2025 Feb 14;15:1478320. doi: 10.3389/fpsyg.2024.1478320 (PMC11869772; doi:10.3389/fpsyg.2024.1478320)
Supplement: Supplementary file 1 [file Presentation_1.pdf]

Supplementary Materials for:

Reciprocal longitudinal effects between sense of school belonging and academic achievement: Quasi-experimental estimates using US primary school data

Chris Sakellariou
Nanyang Technological University, Singapore
acsake@ntu.edu.sg

APPENDIX TABLES AND CHARTS

Table A1: From Social Integration in School to Achievement: First-Stage IV Estimates

	READING		MATHEMATICS	
Sense of School Belonging at grade 4 (stand.)	MALE	FEMALE	MALE	FEMALE
Sensitive to others' feelings	***0.184*** *(0.021)*	***0.131*** *(0.029)*	***0.185*** *(0.021)*	***0.132*** *(0.029)*
Respects others' property	***0.137*** *(0.028)*	***0.144*** *(0.043)*	***0.136*** *(0.028)*	***0.145*** *(0.043)*
Race/Ethnicity: Black	**0.216** (0.065)	0.078 (0.079)	**0.223** (0.064)	0.078 (0.079)
Race/Ethnicity: Hispanic	0.052 (0.053)	**0.133** (0.052)	0.054 (0.053)	**0.136** (0.052)
Race/Ethnicity: Asian	-0.009 (0.078)	-0.115 (0.108)	-0.008 (0.079)	-0.115 (0.108)
Race/Ethnicity: Native American	-0.017 (0.115)	0.059 (0.148)	-0.015 (0.117)	0.058 (0.148)
Mixed Race/Ethnicity	0.099 (0.097)	-0.163 (0.100)	0.100 (0.096)	-0.164 (0.100)
Suburb school (vs. City)	-0.021 (0.041)	0.038 (0.039)	-0.014 (0.042)	0.036 (0.039)
Town school (vs. City)	-0.033 (0.057)	**-0.175** (0.065)	-0.027 (0.057)	**-0.178** (0.065)
Rural school (vs. city)	**-0.204** (0.069)	**-0.131** (0.054)	**0.200** (0.069)	**-0.133** (0.055)
Number of siblings	0.027 (0.015)	-0.002 (0.017)	0.028 (0.015)	-0.001 (0.017)
Has disability	**-0.223** (0.054)	**-0.342** (0.059)	**-0.223** (0.053)	**-0.342** (0.059)
SES (stand. index)	0.039 (0.027)	**0.107** (0.029)	0.039 (0.027)	**0.106** (0.029)
Catholic school (vs. public)	-0.005 (0.087)	-0.088 (0.063)	-0.006 (0.087)	-0.089 (0.063)
Other private (vs. public)	**-0.218** (0.087)	-0.107 (0.104)	**-0.218** (0.087)	-0.107 (0.104)
25-50% non-White (vs. 0-25%)	**-0.156** (0.057)	**-0.137** (0.053)	**-0.157** (0.057)	**-0.138** (0.053)
50-75% non-White (vs. 0-25%)	**-0.286** (0.059)	**-0.117** (0.059)	**-0.282** (0.058)	**-0.115** (0.059)
> 75% non-White (vs. 0-25%)	**-0.286** (0.076)	**-0.167** (0.056)	**-0.285** (0.075)	**-0.171** (0.056)
School problems (stand. index)	**0.039** (0.018)	**0.050** (0.019)	**0.041** (0.018)	**0.050** (0.019)
Numbers Reversed ability score at kindergarten	**0.062** (0.018)	**0.099** (0.021)	**0.062** (0.018)	**0.098** (0.021)
Constant	**-1.42** (0.286)	**-2.02** (0.326)	**-1.41** (0.282)	**-2.01** (0.325)
R^2	0.095	0.090	0.095	0.090
F-statistic [p-value]	19.4 [0.000]	18.1 [0.000]	19.9 [0.000]	18.3 [0.000]
N	4,053	3,970	4,052	3,969

Notes: The model controls for grade 5 school fixed effects. Standard errors derived using 80 Jackknife replication weights in parentheses. Bold indicates statistical significance at the 5% level or lower.

Table A2: From Achievement to Social Integration in School: First-Stage IV Estimates

	READING		MATHEMATICS	
IRT Scale Score at grade 4 (stand.)	**MALE**	**FEMALE**	**MALE**	**FEMALE**
Numbers Reversed ability score at kindergarten	***0.376*** *(0.021)*	***0.317*** *(0.017)*	***0.343*** *(0.017)*	***0.344*** *(0.018)*
Dimensional Change Card Sort at kindergarten	***0.083*** *(0.011)*	***0.078*** *(0.010)*	***0.085*** *(0.010)*	***0.077*** *(0.010)*
Race/Ethnicity: Black	-0.073 (0.056)	-0.070 (0.052)	**-0.427** (0.076)	**-0.420** (0.071)
Race/Ethnicity: Hispanic	-0.080 (0.057)	0.000 (0.034)	**-0.160** (0.055)	-0.033 (0.047)
Race/Ethnicity: Asian	0.160 (0.102)	**0.214** (0.049)	0.159 (0.107)	**0.329** (0.053)
Race/Ethnicity: Native American	**-0.331** (0.088)	-0.001 (0.155)	-0.188 (0.136)	-0.030 (0.079)
Mixed Race/Ethnicity	**0.185** (0.064)	0.076 (0.065)	-0.032 (0.068)	0.019 (0.064)
Suburb school (vs. City)	-0.019 (0.036)	-0.013 (0.040)	-0.036 (0.038)	0.048 (0.039)
Town school (vs. City)	0.055 (0.073)	-0.091 (0.068)	0.042 (0.055)	-0.093 (0.066)
Rural school (vs. city)	**-0.167** (0.049)	-0.047 (0.052)	-0.063 (0.044)	-0.023 (0.051)
Number of siblings	**-0.038** (0.018)	**-0.071** (0.013)	-0.014 (0.015)	-0.019 (0.013)
Has disability	**-0.376** (0.052)	**-0.396** (0.044)	**-0.406** (0.045)	**-0.352** (0.051)
SES (stand. index)	**0.240** (0.024)	**0.258** (0.020)	**0.229** (0.029)	**0.233** (0.020)
Catholic school (vs. public)	-0.047 (0.061)	-0.001 (0.048)	-0.059 (0.068)	-0.097 (0.063)
Other private (vs. public)	**-0.154** (0.060)	-0.043 (0.062)	**-0.209** (0.070)	**-0.198** (0.062)
25-50% non-White (vs. 0-25%)	0.039 (0.040)	0.068 (0.037)	-0.037 (0.052)	0.001 (0.046)
50-75% non-White (vs. 0-25%)	**-0.139** (0.068)	-0.031 (0.055)	-0.094 (0.050)	-0.030 (0.053)
> 75% non-White (vs. 0-25%)	-0.114 (0.069)	-0.100 (0.055)	**-0.142** (0.055)	-0.121 (0.064)
School problems (stand. index)	0.032 (0.017)	0.022 (0.017)	0.000 (0.016)	-0.005 (0.016)
Social Skills at kindergarten (Teacher-rated)	**0.154** (0.026)	**0.081** (0.027)	**0.154** (0.022)	**0.096** (0.028)
Constant	**-1.00** (0.136)	**-0.718** (0.147)	**-0.820** (0.130)	**-0.840** (0.154)
R^2	0.387	0.372	0.429	0.384
F-statistic [p-value]	104.5 [0.000]	131.7 [0.000]	99.2 [0.000]	81.5 [0.000]
N	3,449	3,494	3,449	3,494

Notes: The model controls for grade 5 school fixed effects. Standard errors derived using 80 Jackknife replication weights in parentheses. Bold indicates statistical significance at the 5% level or lower.

Table A3: From Grade 4 Social Integration to Grade 5 achievement – Using one instrument at a time

Outcome: Grade 5 IRT Scale Score (stand.)	READING		MATHEMATICS	
	MALE	FEMALE	MALE	FEMALE
Using ‘*sensitive to others’ feelings’*:<br>Grade 4: Sense of Social integration (stand.)	**0.407** (0.085)	**0.582** (0.110)	**0.338** (0.079)	**0.560** (0.121)
Using ‘*respects others’ property’*:<br>Grade 4: Sense of Social integration (stand.)	**0.386** (0.106)	**0.559** (0.155)	**0.364** (0.115)	**0.529** (0.172)

Table A4: From Grade 4 achievement to grade 5 Social Integration – Using only ‘Card Sort’ score

Outcome: Grade 5 Sense of School Belonging (stand)	READING		MATHEMATICS	
	MALE	FEMALE	MALE	FEMALE
Using ‘*sensitive to others’ feelings’*:<br>Grade 5: Sense of IRT Scale Score (stand.)	0.179 (0.096)	0.170 (0.109)	0.182 (0.099)	0.166 (0.106)

Table A5a: Nevo and Rosen (2012) Imperfect IV Bounds – From School Belonging to Reading achievement

Outcome: Reading score	Lower bound CI	Lower bound estimate	Upper bound estimate	Upper bound CI
Males	[0.218	(0.417	0.645)	1.69]
Females	[0.256	(0.570	0.739)	1.99]

Table A5b: Nevo and Rosen (2012) Imperfect IV Bounds – From School Belonging to Math achievement

Outcome: Math score	Lower bound CI	Lower bound estimate	Upper bound estimate	Upper bound CI
Males	[0.163	(0.316	0.359)	1.12]
Females	[0.237	(0.548	0.852)	2.16]

Table A6a: Nevo and Rosen (2012) Imperfect IV Bounds – From Reading achievement to School Belonging

Outcome: Social Integration	Lower bound CI	Lower bound estimate	Upper bound estimate	Upper bound CI
Males	[0.051	(0.135	0.174)	0.243]
Females	[0.051	(0.194	0.213)	0.331]

Table A6b: Nevo and Rosen (2012) Imperfect IV Bounds – From Math achievement to School Belonging

Outcome: Social Integration	Lower bound CI	Lower bound estimate	Upper bound estimate	Upper bound CI
Males	[0.034	(0.147	0.180)	0.264]
Females	[0.048	(0.178	0.195)	0.305]

Table A7a: Conley et al. (2012) Conservate CIs – From School Belonging to Reading achievement

Outcome: Reading score	Lower bound CI	Coeff. Estimate	Upper bound CI
Males	[0.200	(0.407)	0.614]
Females	[0.276	(0.560)	0.850]

Table A7b: Conley et al. (2012) Conservate CIs – From School Belonging to Math achievement

Outcome: Reading score	Lower bound CI	Coeff. Estimate	Upper bound CI
Males	[0.188	(0.357)	0.526]
Females	[0.155	(0.524)	0.894]

Table A8a: Conley et al. (2012) Conservate CIs – From Reading achievement to School Belonging

Outcome: Reading score	Lower bound CI	Coeff. Estimate	Upper bound CI
Males	[0.037	(0.191)	0.346]
Females	[0.001	(0.154)	0.308]

Table A8b: Conley et al. (2012) Conservate CIs – From Math achievement to School Belonging

Outcome: Reading score	Lower bound CI	Coeff. Estimate	Upper bound CI
Males	[0.062	(0.190)	0.319]
Females	[0.023	(0.153)	0.283]

Table A9a: From Grade 4 School belonging to Grade 5 achievement – Only exogenous covariates

Outcome: Grade 5 IRT Scale Score (stand.)	READING		MATHEMATICS	
	MALE	FEMALE	MALE	FEMALE
Grade 4: Sense of Social integration (stand.)	**0.424** (0.086)	**0.555** (0.108)	**0.376** (0.092)	**0.520** (0.119)
Relevance of excluded instrument:				
Shea Partial R-sq. of excluded instrument	0.044	0.025	0.044	0.025
Montiel Olea – Pflueger weak instrument test:				
Effective F-statistic:	66.5	32.3	66.1	32.62
Critical values for 5% worst-case bias:	7.03	6.88	7.00	6.71
Overidentification test of all instruments:				
Hansen's J statistic [p-value]	0.006 [0.940]	0.084 [0.772]	0.078 [0.781]	0.438 [0.508]
Test of endogeneity of social integration:				
Chi-sq. statistic [p-value]	16.64 [0.000]	29.47 [0.000]	13.35 [0.000]	26.01 [0.000]
N	4,221	4,127	4,219	4,126

Notes: The model controls for grade 5 school fixed effects. Standard errors derived using 80 Jackknife replication weights in parentheses. Bold indicates statistical significance at the 5% level or lower.

Table A9b: From Grade 4 achievement to Grade 5 School belonging – Only exogenous covariates

Outcome: Grade 5: Sense of Social integration (stand.)	READING		MATHEMATICS	
	MALE	FEMALE	MALE	FEMALE
Grade 4: IRT Scale Score (stand.)	**0.159** (0.047)	**0.174** (0.052)	**0.173** (0.051)	**0.158** (0.050)
Relevance of excluded instrument:				
Shea Partial R-sq. of excluded instrument	0.184	0.172	0.196	0.193
Montiel Olea – Pflueger weak instrument test:				
Effective F-statistic:	258.3	232.9	276.8	276.1
Critical values for 5% worst-case bias:	7.52	6.64	8.15	6.20
Overidentification test of all instruments:				
Hansen's J statistic [p-value]	0.024 [0.876]	0.004 [0.950]	0.002 [0.963]	0.032 [0.858]
Test of endogeneity of social integration:				
Chi-sq. statistic [p-value]	0.646 [0.422]	4.06 [0.044]	2.17 [0.141]	3.42 [0.064]
**N**	3,895	3,913	3,897	3,913

Notes: The model controls for grade 5 school fixed effects. Standard errors derived using 80 Jackknife replication weights in parentheses. Bold indicates statistical significance at the 5% level or lower.

Graph 1: From Sense of School Belonging to Reading Scale score: Exclusion restriction tests

MALES **FEMALES**

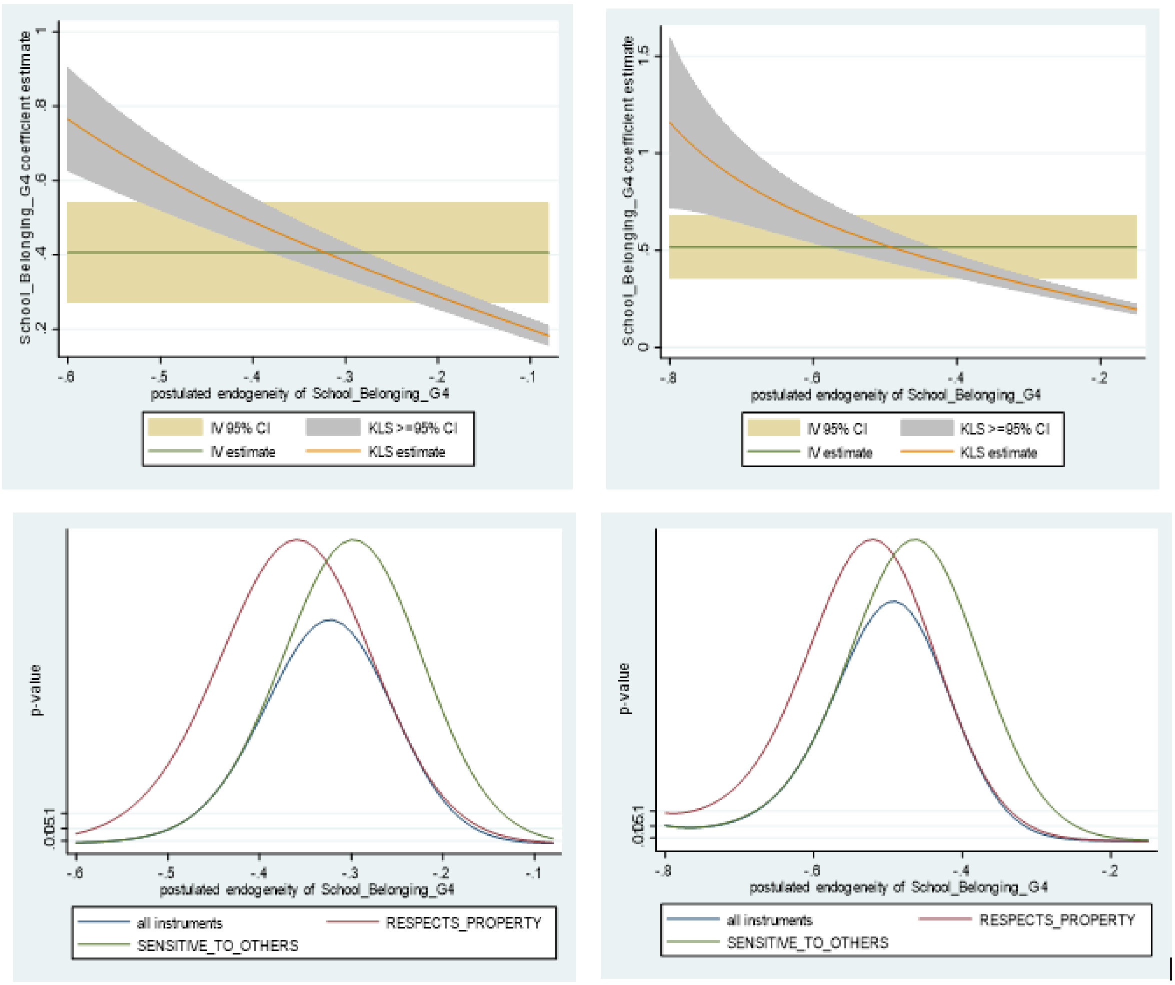

Plausible endogeneity correlations: point estimate [95% CI]

MALES: -0.30 [-0.42, -0.18] FEMALES: -0.45 [-0.57, -0.32]

Degree of endogeneity compatible with valid exclusion: point estimate [95% CI]

Respects others' property:

MALES: -0.36 [-0.58, -0.16] FEMALES: -0.52 [., -0.31]

Sensitive to others' feelings:

MALES: -0.30 [-0.50, -0.11] FEMALES: -0.46 [-0.74, -0.25]

Combination of instruments:

MALES: -0.32 [-0.50, -0.15] FEMALES: -0.49 [-0.80, -0.30]

Graph 2: From Sense of School Belonging to Mathematics Scale score: Exclusion restriction tests

MALES **FEMALES**

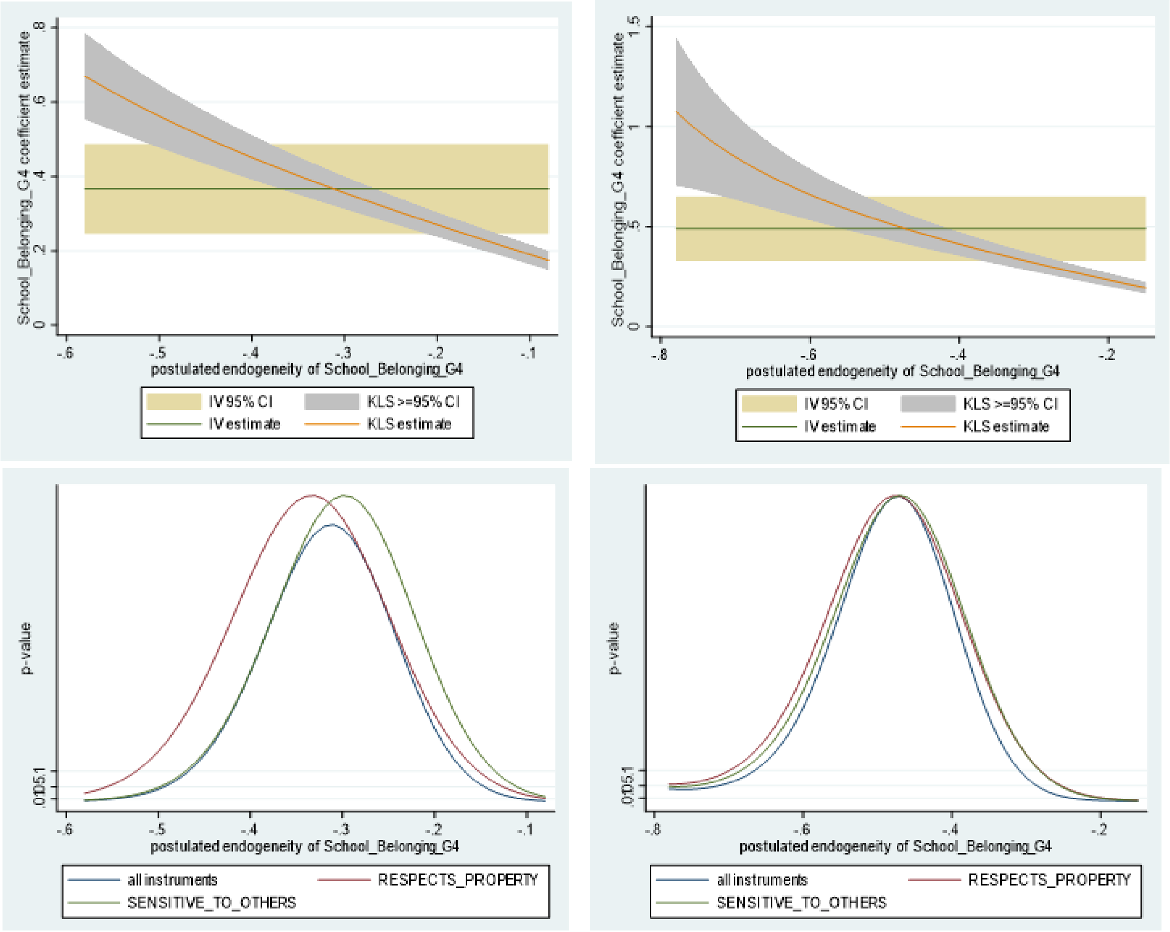

Plausible endogeneity correlations: point estimate [95% CI]

MALES: -0.29 [-0.42, -0.17] FEMALES: -0.44 [-0.56, -0.30]

Degree of endogeneity compatible with valid exclusion: point estimate [95% CI]

Respects others' property:

MALES: -0.33 [-0.56, -0.13] FEMALES: -0.48 [-0 77, -0.25]

Sensitive to others' feelings:

MALES: -0.30 [-0.50, -0.11] FEMALES: -0.47 [-0.75, -0.25]

Combination of instruments:

MALES: -0.30 [-0.50, -0.12] FEMALES: -0.48 [-0.74, -0.29]

Graph 3: From Reading Scale score to Sense of School Belonging: Exclusion restriction tests

MALES **FEMALES**

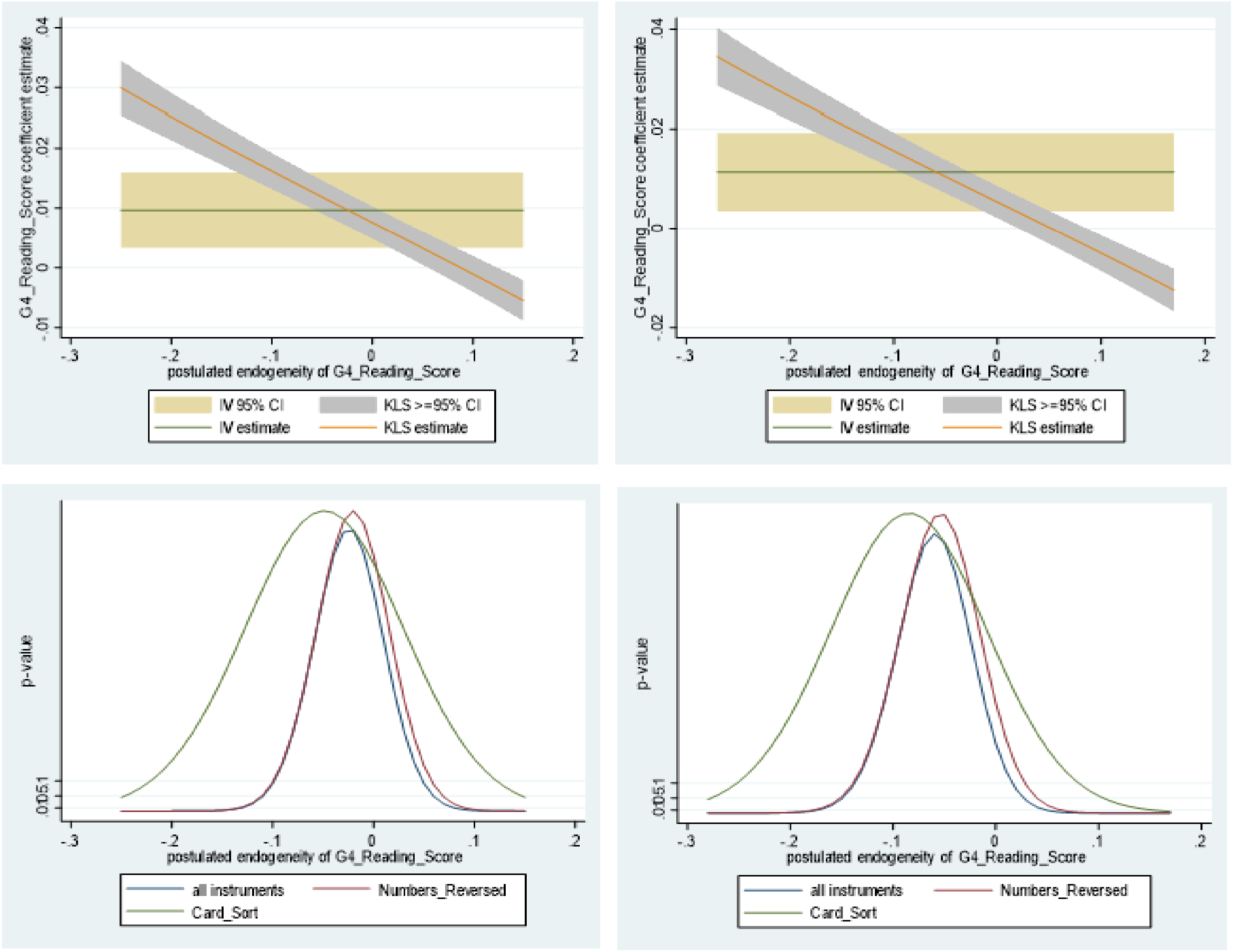

Plausible endogeneity correlations: point estimate [95% CI]

MALES: -0.025 [-0.095, 0.044] FEMALES: -0.062 [-0.13, 0.01]

Degree of endogeneity of instrument compatible with valid exclusion: point estimate 95% CI

Number Reversed score:

MALES: -0.020 [95% CI: -0.11, 0.069] FEMALES: -0.053 [95% CI: -0.15, 0.040]

Dimensional Change Card Sort:

MALES: -0.05 [95% CI: -0.22, 0.15] FEMALES: -0.084 [95% CI: -0.28, 0.10]

Combination of instruments:

MALES: -0.02 [-0.11, -0.05] FEMALES: -0.04 [-0.15, 0.02]

Graph 4: From Mathematics Scale score to Sense of School Belonging: Exclusion restriction tests

MALES | **FEMALES**

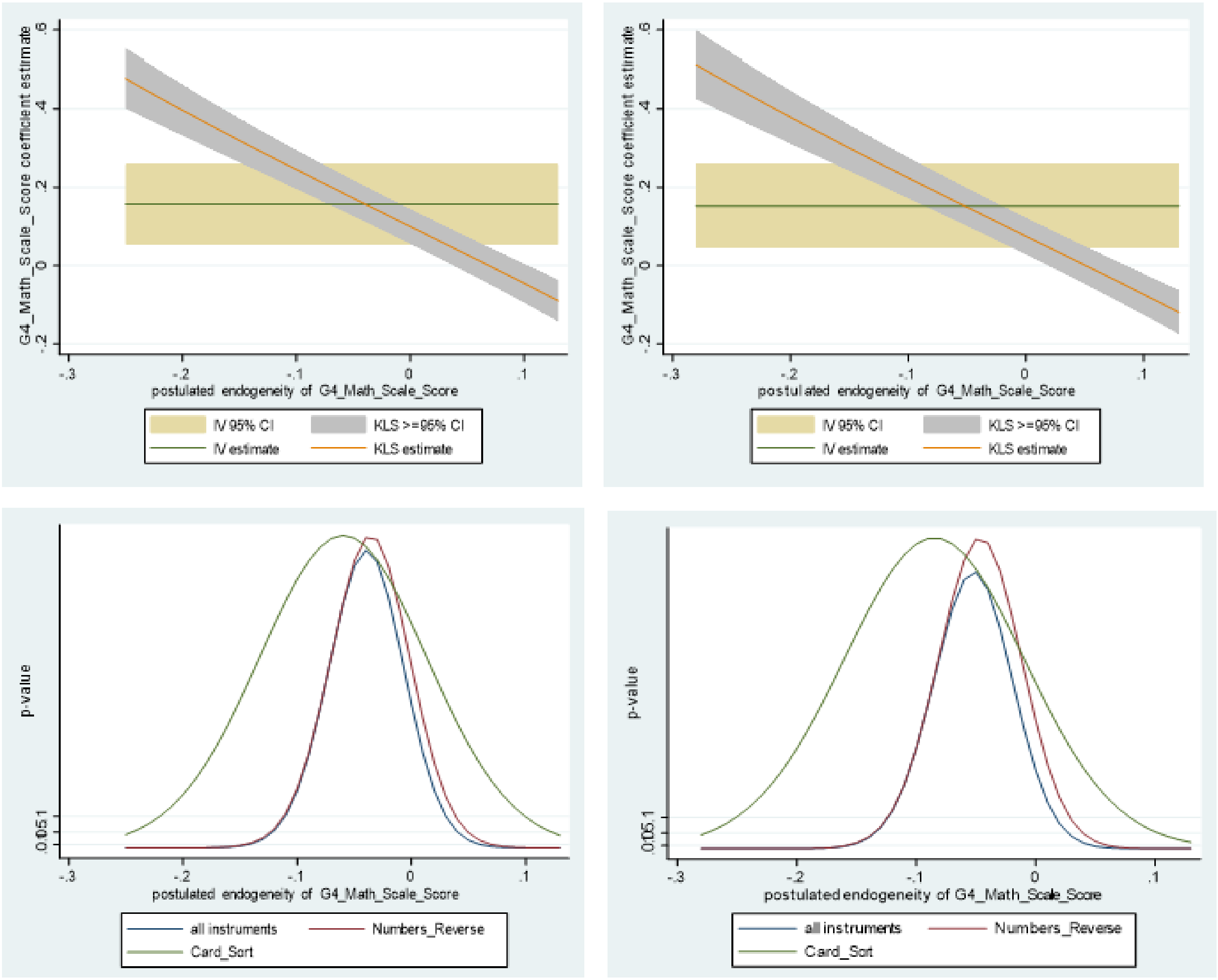

Plausible endogeneity correlations: point estimate [95% CI]

MALES: -0.042 [-0.11, 0.026] FEMALES: -0.057 [-0.13, 0.012]

Degree of endogeneity of instrument compatible with valid exclusion: point estimate 95% CI

Number Reversed score:

MALES: -0.036 [95% CI: -0.12, 0.049] FEMALES: -0.048 [95% CI: -0.14, 0.039]

Dimensional Change Card Sort:

MALES: -0.06 [95% CI: -0.24, 0.12] FEMALES: -0.085 [95% CI: -0.28, 0.10]

Combination of instruments:

MALES: -0.045 [-0.11, 0.03] FEMALES: -0.05 [-0.13, 0.025]
